# Mental Fatigue Alters Cortical Activation and Psychological Responses, Impairing Performance in a Distance-Based Cycling Trial

**DOI:** 10.3389/fphys.2018.00227

**Published:** 2018-03-16

**Authors:** Flávio O. Pires, Fernando L. Silva-Júnior, Cayque Brietzke, Paulo E. Franco-Alvarenga, Fabiano A. Pinheiro, Nanci M. de França, Silmar Teixeira, Tony Meireles Santos

**Affiliations:** ^1^Exercise Psychophysiology Research Group, School of Arts, Sciences and Humanities, University of São Paulo, São Paulo, Brazil; ^2^Human Movement Science and Rehabilitation Program, Federal University of São Paulo, Santos, Brazil; ^3^Brain Mapping and Plasticity Laboratory (LAMPLACE), Federal University of Piauí (UFPI), Parnaíba, Brazil; ^4^Physical Education Program, Catholic University of Brasilia, Brasília, Brazil; ^5^Research Center for Performance and Health, Physical Education Program, Federal University of Pernambuco, Pernambuco, Brazil

**Keywords:** fatigue, motivation, EEG, pacing strategy, prefrontal cortex

## Abstract

**Purpose:** We sought to verify if alterations in prefrontal cortex (PFC) activation and psychological responses would play along with impairments in pacing and performance of mentally fatigued cyclists.

**Materials and Methods:** Eight recreational cyclists performed two preliminary sessions to familiarize them with the rapid visual information processing (RVP) test, psychological scales and 20 km cycling time trial (TT_20km_) (session 1), as well as to perform a VO_2MAX_ test (session 2). Thereafter, they performed a TT_20km_ either after a RVP test (30 min) or a time-matched rest control session (session 3 and 4 in counterbalanced order). Performance and psychological responses were obtained throughout the TT_20km_ while PFC electroencephalography (EEG) was obtained at 10 and 20 km of the TT_20km_ and throughout the RVP test. Increases in EEG theta band power indicated a mental fatigue condition. Repeated-measures mixed models design and *post-hoc* effect size (ES) were used in comparisons.

**Results:** Cyclists completed the trial ~2.7% slower in mental fatigue (34.3 ± 1.3 min) than in control (33.4 ± 1.1 min, *p* = 0.02, very large ES), with a lower W_MEAN_ (224.5 ± 17.9 W vs. 240.2 ± 20.9 W, respectively; *p* = 0.03; extremely large ES). There was a higher EEG theta band power during RVP test (*p* = 0.03; extremely large ES), which remained during the TT_20km_ (*p* = 0.01; extremely large ES). RPE increased steeper in mental fatigue than in control, together with isolated reductions in motivation at 2th km (*p* = 0.04; extremely large ES), felt arousal at the 2nd and 4th km (*p* = 0.01; extremely large ES), and associative thoughts to exercise at the 6th and 16th km (*p* = 0.02; extremely large ES) of the TT_20km._

**Conclusions:** Mentally fatigued recreational cyclists showed impaired performance, altered PFC activation and faster increase in RPE during a TT_20km_.

## Introduction

Mental fatigue is a psychophysiological state caused by a prolonged, high-demanding and sustained cognitive activity that induces a feeling of “tiredness” and “lack of energy” (Boksem and Tops, 2008; Ishii et al., 2014). Although the underlying mechanisms have not been fully elucidated, mental fatigue has been associated with alterations in frontal cortical areas (Lorist et al., 2005; Käthner et al., 2014; Wascher et al., 2014) involved in top-down modulation of behavior (Lorist, 2008). For example, mental fatigue may affect high-order cognitive control (Lorist et al., 2005) and reduce the ability to deal with attentional control, encoding and storage of relevant information, thus leading to a less efficient behavior and greater perceived cost-future reward relationship in a given task (Lorist et al., 2005; Boksem and Tops, 2008; Lorist, 2008; Käthner et al., 2014; Van Cutsem et al., 2017).

Physical performance in self-paced exercises also seems to be related to a correct perceived cost-future reward evaluation and adequate top-down modulation (Smits et al., 2014; Robertson and Marino, 2016). Even in a relatively simple cycling time trial scenario athletes have to use inhibitory control and attentional location on sensory cues while dealing with aversive sensations, in order to adequately regulate their pace and finish the trial as fast as possible (Brick et al., 2016; Martin et al., 2016; Micklewright et al., 2017). In this scenario, mental fatigue may be considered as a threat to a successful performance as this may decrease the ability to deal with aversive sensations and affect the perceived cost-reward relationship during exercise. For example, a recent study reported that recreational, but not professional cyclists slowed down their pace, thereby decreasing the 20 min cycling trial performance when they were mentally fatigued (Martin et al., 2016). These recreational cyclists further rated similar levels of ratings of perceived exertion (RPE) even when the power output was lower during the trial. Ultimately, these results may suggest that mentally fatigued recreational cyclists were more affected by exercise-derived aversive sensations so that they probably decreased performance by facing an increased cost-reward relationship of exercise. Moreover, an impaired inhibitory control (Martin et al., 2016) and attentional location with mental fatigue (Brick et al., 2016) could also be involved in.

Importantly, that mental fatigue study used a time-closed cycling trial, thus probably producing a less realistic scenario when compared to conditions met in cycling training and competitions. Pacing strategy differs between time-closed and distance-closed cycling trials so that cyclists may prefer trials closed by distance in a practical perspective, due to a more realistic approach (Abbiss et al., 2016). The selection of an optimal pacing strategy takes into account the trial distance, rather than duration, as cyclists base their pacing on the perceived distance (Nikolopoulos et al., 2001; Pinheiro et al., 2016). For example, it is proposed that cyclists use a RPE template, created from the momentary RPE in relation to the distance endpoint, to pace themselves during the trial, supporting the notion that the RPE progression may play a role for cycling pacing regulation (de Koning et al., 2011; Schallig et al., 2017). Additionally, the motivation to exercise at high effort levels has also been considered as an important feature to a successful pacing regulation during distance-based trials (Abbiss et al., 2016). As a result, either a RPE progression higher than normal (Van Cutsem et al., 2017) or an insufficient motivation required to overcome exercise-derived aversive sensations (Baron et al., 2011) may be involved in cycling pacing and performance. However, the hypothesis that mentally fatigued recreational cyclists show an impaired pacing regulation and performance, likely together with a faster RPE progression and reduced motivation during a distance-closed cycling trial needs confirmation. Furthermore, as mental fatigue may also impair inhibitory control and attentional location (Boksem and Tops, 2008; Lorist, 2008), one may argue that mentally fatigued recreational cyclists further show less emotional arousal and impaired capacity to allocate attention on internal sensory signals during a distance-based cycling trial.

Alterations in cycling pacing regulation and performance under mental fatigue may be associated with alterations in prefrontal cortex (PFC) activation, as PFC was suggested to be involved in proactive-behavior and goal-directed exercises (Muraven and Baumeister, 2000; Ekkekakis, 2009; Robertson and Marino, 2016). It has been suggested that PFC translates information relative to exercise-induced metabolic disturbances into emotional messages relevant to pacing regulation (Robertson and Marino, 2016). A recent study suggested that endurance regulation in cycling time trial was likely related to the cyclists' ability to preserve motor output despite the reduced PFC activation (Pires et al., 2016). Thus, measures of PFC activation in mentally fatigued recreational cyclists may provide valuable information to understand how mental fatigue may affect pacing and performance. It is important to highlight that studies have suggested that electroencephalography (EEG) theta band measured at PFC may be particularly sensitive to distinguish a mental fatigue condition (Käthner et al., 2014; Wascher et al., 2014), as an increased power of this slow-frequency EEG band suggests a reduced top-down modulation (Lorist, 2008).

Therefore, we verified if mentally fatigued recreational cyclists would show impaired pacing regulation and performance during a distance-based cycling trial. In addition, we verified if alterations in PFC activation and psychological responses would play along with impairments in pacing and performance. Independent studies showed a decreased cycling performance (Martin et al., 2016) and altered PFC activation under mental fatigue (Käthner et al., 2014; Wascher et al., 2014), thus we hypothesized that mental fatigue would impair pacing regulation and performance, and alter PFC activation in a distance-closed cycling trial. Furthermore, we expected that mental fatigue would affect the RPE progression, motivation, emotional arousal, and attention location during the trial.

## Materials and methods

### Participants

Eight recreational, non-professional male cyclists (29.3 ± 7.9 years; 177.2 ± 4.6 cm; 67.6 ± 7.5 kg), with an average of 5.0 ± 3.2 years of experience training and competing at the regional level, volunteered to participate in this study. These cyclists were non-smokers and free from cardiovascular, visual, auditory and cognitive disorders. They were oriented to avoid consumption of stimulant (coffee, energy drink, etc.) and alcoholic beverages, as well as intense exercise for the 48 h preceding the sessions. Experimental procedures, risks, and benefits were explained before collection of their signature on a written consent form. Procedures of this study were approved by a local Ethics Committee (Process: 04254112.9.0000.0029) and performed according to the Declaration of Helsinki.

### Study design

Cyclists attended to four visits during the study: (1ª) to familiarize them with a short version (~5 min) of the rapid visual information processing (RVP) test, psychological scales and a 20 km cycling time trial (TT_20km_); (2ª) to perform an incremental VO_2MAX_ test; (3ª and 4ª) to perform a TT_20km_, either after a 30 min RVP test (experimental session) or after a time-matched rest control session. The first and second sessions were performed sequentially, while the third and fourth sessions were performed in a counterbalanced order, after random designation. All the tests were interspersed by a 3–7 days washout period, performed within 30 days. All experimental procedures were performed at the same time of the day, under controlled temperature (~22°C) and humidity (50–60%). Physiological variables such as EEG and psychological variables such as RPE, motivation, felt arousal scale (FAS) and associative thoughts to exercise (ATE), were measured during TT_20km_. Mood and affect were obtained before and after the RVP test.

Regarding our experimental approach, two aspects should be pointed out. Firstly, different from a recent mental fatigue study using a 20 min time-based cycling trial to investigate cyclists we preferred to use a distance-based trial, as this may represent a more realistic condition met in cycling competitions and training sessions. Moreover, we wanted to use a distance which has already been previously investigated, thus making possible inferences to cycling literature (Silva et al., 2014; Pinheiro et al., 2016). The TT_20km_ filled these requirements, being a long-endurance trial which may potentiate mental fatigue effects on physical performance (Van Cutsem et al., 2017). Secondly, a high-demanding cognitive task with the potential to induce cerebral alterations and mental fatigue (RVP test) was used (Coull et al., 1996; Lawrence et al., 2003; Lim et al., 2010; Hilti et al., 2013). In order to ensure that mental fatigue was induced we assumed that cyclists would have to show an increased PFC EEG theta band power when mentally fatigued, as suggested elsewhere (Sauseng et al., 2010; Käthner et al., 2014). Furthermore, cognitive performance in RVP test and alterations in mood state were used to confirm a mental fatigue state.

### Rapid visual information processing (RVP) test

The RVP test was performed in a comfortable, quiet and illuminated room, while cyclists were placed frontally to a 17 inches colored monitor. The RVP test consisted of 30 min exhibiting numbers (from 1 to 9) in a black-blue box in the center of the monitor, in a random order so that each number were displayed individually (i.e., one by one) at a rate of 100 times per minute. Cyclists were asked to press the space bar of a standard keyboard always when they identified a sequence of three even (e.g., 2, 4, 6; 4, 6, 8) or odd numbers (e.g., 3, 5, 7; 3, 9, 7), shown 8 times a minute. Cognitive performance was measured as false alarms (u.a), reaction time (ms) and accuracy of answers (percentage of numerical sequences wrongly identified). In order to have a control session without negative or positive mental manipulation, cyclists remained resting for 30 min in a comfortable seat in the laboratory. Although no active intervention such as reading a magazine, listening to music, watching a film, etc., was used during this time, no effort was made to create an environment away from a laboratory routine. Cyclists were informed about the objective of the RVP test after they conclude the participation in the study.

### Cycling time trial (TT_20km_)

Cyclists were initially familiarized with the TT_20km_ during a preliminary session, before performing the trial in control and mental fatigue sessions. A road bicycle (SoulCycle®, New York, USA) was attached to a cycle simulator (Computrainer, Racer Mate® 8000, Seattle, USA) which provided power output (W), cadence (rpm) and speed (km.h^−1^) data throughout the test. The device was calibrated before each test according to the manufacturer's instructions. The bicycle was individually adjusted, according to cyclists' preferences.

After a standard 7 min warm-up consisting of a 5 min self-paced warm up (gear and pedal cadence freely adjusted) followed by a 2 min controlled-pace warm up (cycling at a power output of 100 W and pedal cadence of 80 rpm), they immediately started the TT_20km_. Cyclists were oriented to finish the trial within the shortest possible time (sat down throughout the trial). They were free to pace themselves in all the sessions, using distance and elapsed time as a feedback. Due to experimental procedures involving the completion of the questionnaire and scale, and the EEG check, the TT_20km_ started ~10 min after the RVP test execution in mental fatigue session. Accordingly, the control TT_20km_ was initiated ~10 min after the 30 min rest control period.

### Procedures, measures, and data analysis

#### Performance

The time to complete the TT_20km_ and the mean power output (W_MEAN_) recorded throughout the trial were used as performance measures. Power output was further averaged every 2 km in order to analyze pacing strategy.

#### Electroencephalography (EEG)

PFC activation was continuously obtained by using an EEG unit (NeuroSpectrum-5, Neurosoft®, Ivanovo, Russia) with a 500 Hz sampling frequency. Active electrodes (Ag-AgCl) with resistance ~5 KΩ were placed on the scalp, at the FP1 position, according to the international EEG 10–20 system. This position was ensured according to frontal and sagittal planes, referenced to mastoid. After exfoliation and cleaning, electrodes were fixed with a conductive gel, adhesive tape, and medical strips. Then, EEG obtained at the FP1 position during 3 min baseline, throughout the RVP task, and during TT_20km_ was analyzed to represent activation in PFC. In order to reduce artifacts, adhesive tape was used to fix the EEG unit's cables to the individuals' trunk. Importantly, cyclists were familiarized to be accustomed to keeping their eyes opened and avoid jaw movements throughout the EEG measurements during baseline, RVP test, and TT_20km_. They were further familiarized to maintain upper limbs, head, and neck as steady as possible during a 15 s period at the 10th and 20th km of the TT_20km_. These procedures allowed a reasonable EEG signal at 50 and 100% of the cycling trial. Cases showing spectral leakage (assumed as a signal ± 100 μv) were considered as excessive artifacts (*n* = 1–2, depending on the part of the setup).

The surface signal was amplified (gain of 1.000) and treated with a notch (60 Hz) and a 1–30 Hz bandpass filter. The data collected during the RVP test as well as during the cycling trial was normalized to the signal captured between 120 and 180 s of the baseline period. Thereafter, the EEG data were analyzed in frequency domains through a fast-Fourier transformation. A Blackman window, having a zero padding, was applied to avoid frequency leakage and obtain a power spectrum frequency resolution of 0.2 Hz. The area under the theta band power spectrum (3–7 Hz) was calculated over 15 s windows as previous EEG studies have suggested that EEG theta band (a slow-frequency EEG band) is sensitive to distinguish a mental fatigue state (Smith et al., 2003; Sauseng et al., 2010; Käthner et al., 2014; Wascher et al., 2014). Thus, the EEG obtained throughout the RVP test was analyzed at 10, 20 and 30 min, and the EEG obtained during exercise was analyzed at 10th and 20th km of the TT_20km_.

#### Psychological responses

Cyclists completed the mood and affect questionnaires before and immediately after the RVP test. Briefly, a shortened version of the profile of mood states (POMS) questionnaire composed of 24 single-word mood descriptors was used to measure anger, confusion, depression, fatigue, tension, and vigor through a 5-points Likert scale ranging from 0 (zero meaning “nothing”) to 4 (meaning “extremely).” Previous studies have applied this questionnaire in physical exercise approaches (Viana et al., 2016), reporting large internal consistency for these subcategories (values expressed as Cronbach's alpha >0.70) The total mood disorder (TMD) was calculated by summing subcategories such as anger, mental confusion, depression, fatigue, and tension, thereafter subtracting them from vigor (TDM was obtained adding 100 to the final value). Increases in TMD can be interpreted as a decreased mood state and decreases in TMD may be interpreted as an increased mood state. Cyclists also completed the positive and negative affect schedule questionnaire, which consists of a 10-item scale providing independent measures of positive and negative affect (Carvalho et al., 2013). This questionnaire uses a 5-point Likert scale so that 1 corresponds to “no or very little” while 5 corresponds to “very.”

Psychological responses (i.e., RPE, motivation, FAS, and ATE) were assessed every 2 km of the TT_20km_. The RPE was obtained through a 15-points Borg scale (Borg, 1982) as suggested elsewhere (Borg, 1982; Pires et al., 2011). In order to have comparisons with previous studies (Pinheiro et al., 2016; Viana et al., 2016) the RPE_SLOPE_ was calculated (as a function of the distance), thus indicating the rate of linear increase in RPE. Furthermore, motivation was assessed through a 5-points Likert scale with two opposite motivational descriptors, that is “very unmotivated” and “highly motivated.” This Likert scale is similar to that used in previous studies (Smirmaul et al., 2015).

The felt arousal was obtained through the 6-points FAS (Svebak and Murgatroyd, 1985) which classifies arousal within categories ranging from “low activation” to “high activation.” The perception of high arousal may be interpreted as a state of “worked-up,” while the perception of low arousal may be interpreted as a feeling of “relaxation” (Svebak and Murgatroyd, 1985). Additionally, ATE was measured on a bipolar Likert scale with ATE ranging from 0 to 100%. Cyclists rated the ATE based on their internal cues, that is the sensations related to body signals such as sweating, heart rate, breathing and muscle discomfort. In contrast, dissociated thoughts were unrelated to body sensations, thus normally associated with daily tasks such as day-dreaming, personal projects, life, environment, etc. For example, ATE measures close to 0–10% would suggest thoughts highly dissociated from the exercise, otherwise, measures close to 90–100% may suggest thoughts highly related to the exercise (Tammen, 1996; Razon et al., 2009). Although being aware of associated and dissociated thoughts, only ATE were reported. Validity of this ATE scale has been indicated elsewhere (Razon et al., 2009).

### Statistical analysis

Gaussian distribution and homoscedasticity were ensured through Shapiro-Wilk and Levene tests, respectively. Effects of RVP test were checked in two ways. Firstly, alterations in mood and affect from pre to post RVP test were verified by using a Wilcoxon test. Secondly, RVP test effects were verified by comparing false alarms, reaction time, accuracy and EEG responses during RVP test through a number of mixed models, having time (i.e., 10, 20, and 30 min) and mental state (i.e., control and mental fatigue) as the fixed factors, and cyclists as the random factor. The best repeated-measures covariance structure fitting the dataset was obtained among a number of structures, such as Compound Symmetric, First-order Autoregressive (homogeneous and heterogeneous), First-order Autoregressive Moving Average and Toeplitz (homogeneous and heterogeneous). Multiple comparisons were corrected through the Bonferroni's test in cases of significant *F*-values.

We compared the time to complete TT_20km_ and W_MEAN_ with a paired t-Student test. Furthermore, power output and psychological responses (i.e., RPE, motivation, FAS and ATE) during the TT_20km_ were compared through a number of mixed models, having distance (i.e., 2nd, 4th up to 20th km) and mental state (i.e., control and mental fatigue) as the fixed factors, the cyclists were the random factor. Accordingly, EEG responses at 10 and 20 km of the TT_20km_ were analyzed through a mixed models comparison. In these analyzes, we further used the best covariance structure fitted to the dataset, calculated among different structures (Compound Symmetric, First-order Autoregressive homogeneous and heterogeneous, First-order Autoregressive Moving Average, and Toeplitz homogeneous and heterogeneous). Multiple comparisons were corrected by Bonferroni test in cases of significant *F*-values. The RPE_SLOPE_ was compared between control and mental fatigue by a paired t-Student test. Importantly, as we did not perform a prior sample size calculation, we calculated the effect size (ES) as a *post-hoc* analysis for every significant result, using the appropriate index for t-Student test, mixed models or non-parametric analysis. In order to make the interpretation of different ES indexes easier for the reader, we classified them as small, moderate, large, very large and extremely large, similar to suggested elsewhere (Hopkins et al., 2009). Statistical power was >0.80 for all analysis, and significant results were accepted if *p* < 0.05. Results were reported as the mean and standard deviation (± SD).

## Results

As a control of the study, we verified if there would have been any order effect in performance responses. Comparisons showed no difference between the first and second TT_20km_ session, neither for time to complete the trial (33.9 ± 1.7 min vs. 33.8 ± 0.7 min; *p* = 0.77) nor for W_MEAN_ (225.7 ± 36.0 W vs. 226.2 ± 23.4 W; *p* = 0.97). Cyclists attained a peak power output of 318.9 ± 22.4 W and a VO_2MAX_ of 64.1 ± 4.8 ml·kg·min^−1^ during the preliminary incremental VO_2MAX_ test.

### Effects of RVP test on cognitive performance, EEG and psychological responses

Overall results showed that a 30 min RVP test induced mental fatigue. There was an impairment in cognitive performance as RVP test progressed, since false alarms increased [10th min = 29.7 ± 27.9, 20th min = 38.7 ± 31.6, 30th min = 44.4 ± 43.6, *F*_(4, 69)_, *p* = 0.03, η^2^ = 0.25, extremely large ES] and accuracy decreased [10th min = 11.5 ± 9.2%, 20th min = 11.4 ± 6.2%, 30th min = 15.5 ± 7.8%, *F*_(3, 86)_, *p* = 0.04, η^2^ = 0.22, extremely large ES] from 10 to 30 min. No change was found in reaction time (*p* > 0.05).

**Figure 1:**
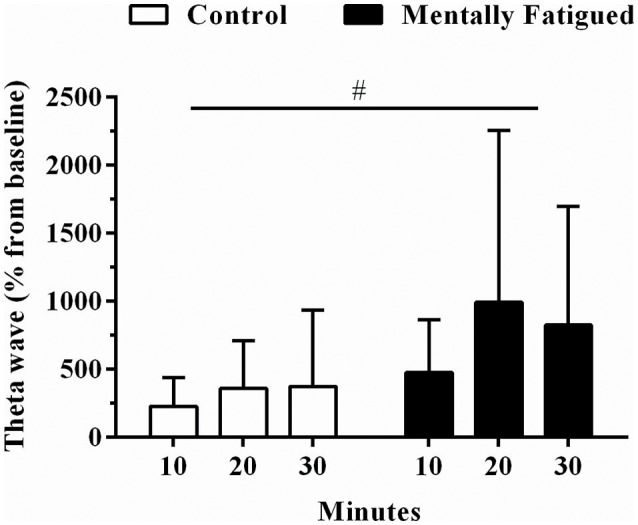
Data were reported as mean ± SD. Fp1 theta power responses during rapid visual information processing test. Open and filled boxes are control and mental fatigue condition, respectively. ^#^Mental fatigue main effect (*p* = 0.03, ES = extremely large).

The RVP test also induced changes in EEG, as the PFC theta band power increased as the RVP progressed. Multiple comparisons detected a mental fatigue main effect [*F*_(5, 81)_, *p* = 0.03, η^2^ = 0.3, extremely large ES] so that Fp1 theta power recorded at 10, 20, and 30 min was higher than matched-time control values. Additionally, neither time main effect (*p* > 0.05) nor mental fatigue by time interaction effect (*p* > 0.05) was observed in Fp1 theta power (Figure 1).

As a result of the RVP test, there was a decrease in positive (Pre = 21.3 ± 5.39 to Post = 16.6 ± 4.1, *Z* = −2.26, *p* = 0.02; extremely large ES) and an increased in negative affect (Pre = 13.7 ± 4.3 to Post = 22.2 ± 6.5, *Z* = −2.39, *p* = 0.03; extremely large ES). Accordingly, mood responses were impaired as there was a decrease in vigor (*Z* = −2.64, *p* = 0.01; extremely large ES) and an increase in tension (*Z* = −2.23, *p* = 0.03; extremely large ES) and mental confusion (*Z* = −2.37, *p* = 0.02; extremely large ES), thus resulting in a greater TMD (*Z* = −2.54, *p* = 0.01, extremely large ES). Other POMS subscales such as depression, anger and fatigue were not affected by RVP test (*p* > 0.05). When comparing time-matched responses between mental fatigue and control sessions, that is mood responses after the RVP test between mental fatigue and control, we observed a greater TMD in mental fatigue session (*Z* = −2.26, *p* = 0.02; extremely large ES). Table 1 shows these results.

**Table 1:** Profile mood state responses before and after the RVP test (mental fatigue condition) or 30 min rest (control condition).

		**Control**	**Mentally fatigued**
Tension	Pre	2.1 ± 2.1	1.1 ± 1.7
	Post	1.8 ± 3.0	2.7 ± 3.3^#^
Depression	Pre	0.7 ± 1.1	0.8 ± 1.4
	Post	1.2 ± 2.7	1.5 ± 2.7
Anger	Pre	0.6 ± 1.4	0.6 ± 1.4
	Post	1.3 ± 3.5	1.5 ± 3.1
Vigor	Pre	9.2 ± 2.8	9.2 ± 2.0
	Post	7.0 ± 4.1^‡^	5.3 ± 2.3^**^
Fatigue	Pre	1.3 ± 1.1	1.8 ± 1.3
	Post	2.6 ± 3.0	3.5 ± 2.8
Mental confusion	Pre	0.6 ± 1.4	1.0 ± 1.9
	Post	1.7 ± 3.4	3.2 ± 3.4^*^
TMD	Pre	96.6 ± 5.1	96.25 ± 6.4
	Post	102.2 ± 14.8	106.7 ± 13.2^†$^

Data were reported as mean ± SD. TMD is total mood disorder. Comparisons between conditions:

# Z = −2.23, p = 0.03. Comparisons between pre and post moments:

** Z = −2.64, p = 0.01;

‡ Z = −2.23, p = 0.03;

* Z = −2.37, p = 0.02;

†$ *Z = −2.54, p = 0.01*.

### Effects of RVP test on TT_20km_ performance and pacing

**Figure 2:**
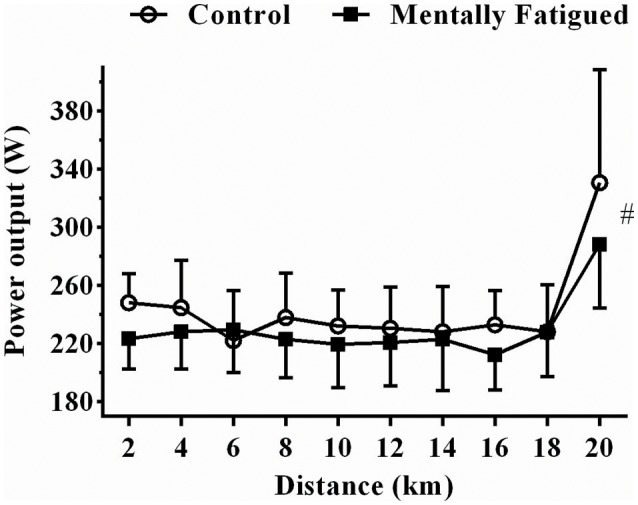
Data were reported as mean ± SD. Power output during TT_20km_ in control (open circles) and mental fatigue condition (filled boxes). ^#^Mental fatigue main effect (*p* = 0.03, ES = extremely large) (distance main effects have been suppressed for a better view of the mental fatigue effects; readers are referred to the Results section).

The TT_20km_ performance was significantly impaired when cyclists were mentally fatigued, as the time to complete the trial (34.3 ± 1.3 min) was ~ 2.7% slower in mental fatigue than in control session (33.4 ± 1.1 min) (*t* = −3.14, *p* = 0.02, *d* = 0.74, very large ES). Accordingly, W_MEAN_ was reduced ~6.5% (*t* = 2.78, *p* = 0.03, *d* = 0.98, extremely large ES) in mental fatigue (224.5 ± 17.9 W) when compared to control (240.2 ± 20.9 W). Cyclists adopted a “j-shaped” pacing strategy during the TT_20km_ in both condition, thus power output values from the 18th km was greater than values from previous distances [*F*_(21, 11)_, *p* = 0.001, η^2^ = 0.12, very large ES; Figure 2].

#### Effects of RVP test on EEG and psychological responses during TT_20km_

**Figure 3:**
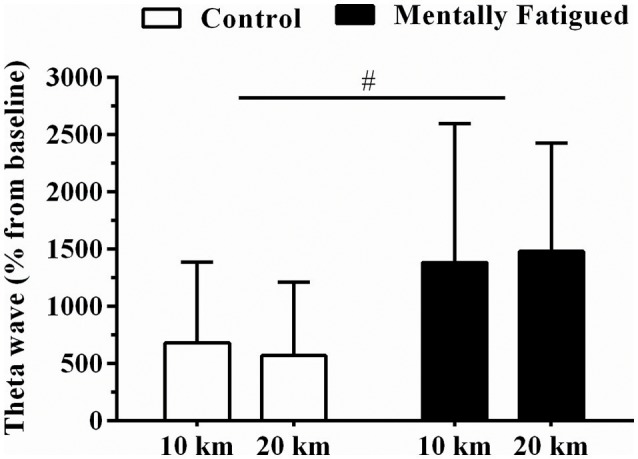
Data were reported as mean ± SD. EEG theta band measured at PFC (prefrontal cortex) during TT_20km_ in control (open boxes) and mental fatigue condition (filled boxes) ^#^Mental fatigue main effect (*p* = 0.01, ES = extremely large).

Regarding EEG measures, mentally fatigued recreational cyclists showed higher Fp1 EEG theta band power [*F*_(5, 78)_, *p* = 0.01, η^2^ = 0.29, extremely large ES] in mental fatigue than in control. However, neither distance main effect (*p* > 0.05) nor mental fatigue by distance interaction effect (*p* > 0.05) was observed (Figure 3).

**Figure 4:**
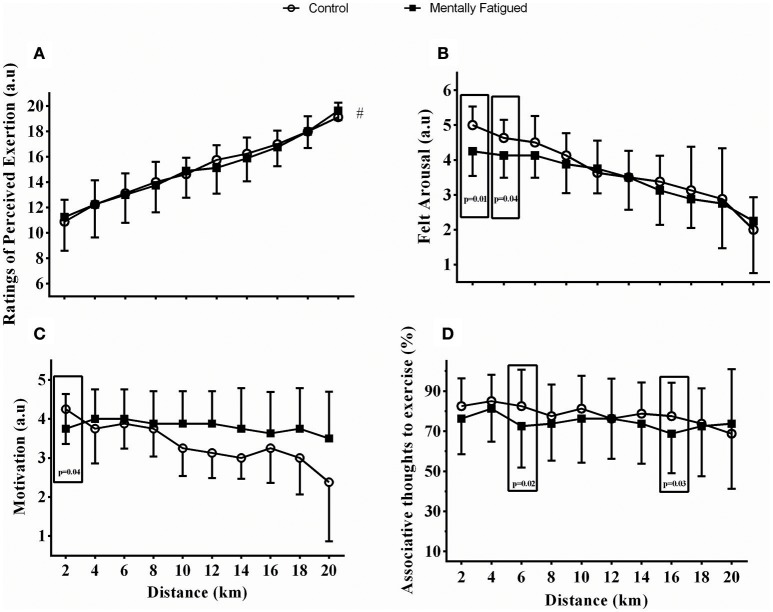
Data were reported as mean ± SD. Ratings of perceived exertion **(A)**, Felt arousal **(B)**, Motivation **(C)**, and Associative thoughts to exercise **(D)** responses during TT_20km_ in control (open circles) and mental fatigue condition (filled boxes). Distance by mental fatigue interaction effects were highlighted in squares (distance main effects have been suppressed for a better view of the mental fatigue effects; readers are referred to the Results section). ^#^Difference between mental fatigue and control SLOPE (*p* = 0.002, ES = extremely large).

Regarding psychological responses, a distance main effect was observed in RPE [*F*_(73, 90)_, *p* < 0.0001, η^2^ = 0.84; extremely large ES], motivation [*F*_(5, 08)_, *p* < 0.001, η^2^ = 0.26; extremely large ES] and FAS [*F*_(24, 97)_, *p* < 0.0001, η^2^ = 0.64, extremely large ES], but not in ATE (*P* > 0.05), thereby indicating a progressive change in most psychological responses as the trial progressed. Although no mental fatigue main effect has been detected (*p* > 0.05), there was a distance by mental fatigue interaction effect since motivation was lower at 2 km [*F*_(2, 65)_, *p* = 0.04, η^2^ = 0.36, extremely large ES], FAS was lower at 2 and 4 km [*F*_(4, 58)_, *p* = 0.01, η^2^ = 0.69, extremely large ES] and ATE was lower at 6 and 16 km [*F*_(3, 05)_, *p* = 0.02, η^2^ = 0.56, extremely large ES] in mental fatigue. Accordingly, mental fatigue speeded up the RPE increase (*t* = −2.736, *p* = 0.002, *d* = 0.98; extremely large ES), as RPE_SLOPE_ was greater in mental fatigue (0.4 ± 0.1 a.u·km^−1^) than in control (0.3 ± 0.1 a.u.·km^−1^). Figures 4A–D shows these results.

## Discussion

This study showed that mental fatigue impaired cycling performance without changing pacing strategy in a distance-based cycling trial, as we observed that mentally fatigued recreational cyclists impaired the TT_20km_ performance by ~2.7% (~1 min), but conserved a J-shaped pacing profile in both conditions. Further, we observed that these mentally fatigued cyclists showed a change in PFC activation, perhaps related to changes in psychological responses.

The present study provides insightful information with regard to mental fatigue effects on physical performance. It has been well documented that attentional tasks requiring executive functions such as alternating attention, goal-directed attention, sustained attention, response inhibition and working memory can overload cerebral areas involved in high-order cognitive control, thus impairing top-down modulation (Lorist et al., 2005; Lorist, 2008; Ishii et al., 2014). Hence, alterations in frontal cortical areas such as PFC are expected to occur if a high-demanding attentional task progresses (Käthner et al., 2014; Wascher et al., 2014). Thus, similar to results reported elsewhere (Käthner et al., 2014; Wascher et al., 2014) we also observed increased Fp1 EEG theta power as RVP test progressed. Interestingly, such an increase in Fp1 EEG theta power remained during the TT_20km_. Therefore, alterations in PFC activation during TT_20km_ were likely a result of mental fatigue, rather than of changes in power output, as alterations in EEG theta band were readily observed during RVP test.

Furthermore, mentally fatigued recreational cyclists showed an impaired TT_20km_ performance, thereby corroborating a possible connection between physical performance and changes in PFC activation (Pires et al., 2016; Robertson and Marino, 2016). It has been proposed that PFC plays a key role in pacing and exercise regulation, as PFC is involved in proactive, goal-directed behavior (Miller and Cohen, 2001; Ekkekakis, 2009). Studies have suggested that successful self-paced exercise performance is related to superior inhibitory control (Muraven and Baumeister, 2000; Martin et al., 2016) and attentional location (Brick et al., 2016) so that the increased slow-frequency EEG activity in mentally fatigued cyclists could reflect their lower ability to preserve adequate inhibitory control and attentional location during exercise. Consequently, they may have had less cognitive ability to deal with aversive feelings while they had to self-regulate pacing (Micklewright et al., 2017).

We have hypothesized that changes in PFC activation may indicate impaired top-down modulation, thereby influencing psychological responses such as RPE, motivation, emotional arousal and attention location. Previous studies observed that the linear increase in RPE was greater in mentally fatigued individuals either in a controlled-pace cycling (Marcora et al., 2009) or in a self-paced running (Pageaux et al., 2014), therefore indicating that the perceived exertion was higher than normal in mentally fatigued individuals. Accordingly, we observed that RPE increased linearly throughout the TT_20km_ in both conditions, but the greater RPE_SLOPE_ in mentally fatigued cyclists indicated that mental fatigue speeded up the linear increase in RPE. Importantly, as this greater RPE_SLOPE_ was observed with a lower W_MEAN_ in mental fatigue, cyclists were likely less resistant to exercise when they were mentally fatigued.

Alterations in other psychological responses were less evident, as most were observed only within the first 6 km of the TT_20km_ (i.e., a mental fatigue by distance interaction effect). Somehow, these results may suggest that mentally fatigued recreational cyclists were less cognitively resourceful to start a goal-driven, motivational-behavior exercise focused on pace-related thoughts such as memory and attention location (Miller and Cohen, 2001; Ekkekakis, 2009; Martin et al., 2016; Micklewright et al., 2017). In this sense, cyclists may have shown less ability to access attention location at the first stages of the TT_20km_ (Miller and Cohen, 2001; Braver, 2012), as the lower FAS could indicate impaired vigilance sustained-attention (Oliveira et al., 2013) and the lower ATE, an inadequate attention on internal sensory monitoring (Razon et al., 2009; Pinheiro et al., 2016). However, this suggestion should be interpreted with caution, as the absence of a mental fatigue main effect could indicate an accidental, rather than a systematic mental fatigue effect. Future studies are required to confirm this suggestion.

### Practical implications and methodological aspects

Instead of using a time-based cycling trial (Martin et al., 2016), we used a mental fatigue paradigm in a distance-based cycling trial, a laboratory trial (i.e., TT_20km_) traditionally used in scientific investigations (Silva et al., 2014; Pinheiro et al., 2016). We preferred a trial closed by distance as this may represent a more realistic condition met in cycling competitions and training sessions (Abbiss et al., 2016). Normally, cyclists take into account the perceived trial distance, rather than duration, when selecting an optimal pacing strategy (Nikolopoulos et al., 2001; Pinheiro et al., 2016). Consequently, cyclists performed a J-shaped pacing strategy and spurted at the end of the distance-based trial, as they were allowed to refer to the available distance feedback to base their perceived distance during the trial. Hence, similar to results reported in running (Pageaux et al., 2014) we observed that prior high-demanding cognitive task (RVP test) led to a significant decrease in W_MEAN_ and time to complete the cycling trial, without changing the J-shaped pacing strategy yet. Therefore, supporting this previous running study (Pageaux et al., 2014) these results showed that mental fatigue impaired the TT_20km_ performance without changing the pacing profile of recreational cyclists. However, we must highlight that the robust pacing profile observed in these mental fatigue studies may have been a result of the available distance feedback (Smits et al., 2016), so that future studies are required to verify how mental fatigue may affect pacing regulation when the feedback of distance is unavailable. Importantly, the present results provided insights into how mental fatigue may impact cycling pacing and performance in recreational cyclists, as most of them combine high-load aerobic training programs with a strict-life style (food intake, alcohol consumption, etc.) and daily activities (e.g., driving or moving through a busy city, dealing with financial life, accumulating different jobs, etc.).

Some aspects regarding EEG measures should be pointed out. Firstly, recent study verified that mentally fatigued individuals showed an increased PFC beta power when they were submitted to a RPE-matched exercise, and this increased EEG beta power was interpreted as an indication of mental fatigue in that study (Brownsberger et al., 2013). However, an increase in theta power rather than in beta power is suggested to reflect mental fatigue in neuroscience literature (Käthner et al., 2014; Wascher et al., 2014). Thus, we have used a standard slow-frequency EEG band to confirm mental fatigue, that is, an increase in PFC theta power. In addition, we also confirmed a mental fatigue condition by reductions in mood and affect responses as well as impairments in cognitive performance during RVP test. Therefore, instead of isolated mood and cognitive performance measures as traditionally reported (Van Cutsem et al., 2017), we used a complete scenario to ensure that cyclists were mentally fatigued.

Secondly, the use of EEG technique to monitor changes in cortical activation during exercise has been criticized, as artifacts derived from upper body movement can impair EEG analysis (Thompson et al., 2008). During the experimental setup, we used active electrodes, fixed cables, and electrodes, and familiarized cyclists to keep their eyes opened without jaw movements, while maintaining upper limbs as steady as possible during EEG measures. Although carefully controlling our experimental setup, we acknowledge that artifacts associated with whole-body exercises may challenge the EEG interpretation. Apparently, this is the first study providing EEG measures during cycling trial in mentally fatigued recreational cyclists, future studies are encouraged to improve the use of EEG measures in whole-body exercises.

## Conclusion

The present study showed that TT_20km_ performance was impaired when recreational cyclists were mentally fatigued, although a J-shaped pacing strategy was conserved in this distance-based cycling trial. Furthermore, PFC activation was changed and RPE increased faster in mental fatigue, probably playing along with this impaired cycling performance.

## Author contributions

All authors contributed to this study. FOP, FS-J, and TMS conceived and designed the study. FOP and TMS provided funding. FS-J and FAP collected data. FOP, FAP, CB, PF-A, and ST analyzed and interpreted data. FOP, FAP, CB, PF-A, ST, and TMS wrote and FAP, CB, PF-A, NF, ST, and TMS reviewed the manuscript.

### Conflict of interest statement

The authors declare that the research was conducted in the absence of any commercial or financial relationships that could be construed as a potential conflict of interest.
